# Local excision as a treatment for tumors of ampulla of Vater

**DOI:** 10.1186/1477-7819-4-14

**Published:** 2006-03-08

**Authors:** Haralampos Demetriades, Emmanouil Zacharakis, Ioanna Kirou, Manousos-Georgios Pramateftakis, Nikolaos Sapidis, Ioannis Kanellos, Dimitrios Betsis

**Affiliations:** 1 4^th ^Surgical Department. Aristotle University of Thessaloniki, 'G. Papanikolaou' General Hospital, Exohi, Thessaloniki 57010, Greece

## Abstract

**Background:**

Although local excision (ampullectomy) was first described by Halsted in 1899, its adequacy as an alternative surgical treatment for the ampullary tumors is still a matter of debate. The aim of this study was to evaluate the results of ampullectomy as a curative treatment for benign and malignant tumors arising from the ampulla, in a 14-year single-institution experience.

**Methods:**

From 1990 to 2004, a total of 20 patients of adenocarcinoma (12) or adenoma (8) of the ampulla of Vater underwent local excision. Clinical data were collected and morbidity, mortality, as well as long-term survival were evaluated. The usefulness of several pre or intraoperative diagnostic methods was also recorded. Median follow-up was 85 (range 6–180) months.

**Results:**

The combination of endoscopic preoperative biopsies and intraoperative frozen section examination adequately diagnosed ampullary tumors in all cases. The postoperative morbidity and mortality were 0%, whereas the 3 and 5-year survival rates for the patients with adenocarcinoma was 75 % and 33.3 % respectively. All the patients with adenoma are still alive without any sign of recurrence.

**Conclusion:**

In our series, local excision was a safe option, associated with satisfactory long-term survival rates in patients with benign lesions and in those with small(<2 cm), pT1, well differentiated ampullary tumours without nodal involvement.

## Background

Mass lesions of the ampulla of Vater represent less than 10% of pancreatic and periampullary tumors. Among these, carcinomas, adenomas and neuroendocrine tumors are the most frequently recognized neoplasms [1].

Since 1935, pancreatoduedectomy (PD) has been used as the treatment of choice for the neoplasms arising from the ampulla of Vater [2-4]. Although mortality rates have been reduced nowadays, PD is still associated with high morbidity rates [3,5-7]. In order to reduce PD associated complications, local excision of the ampulla of Vater (ampullectomy) has been introduced as an alternative procedure for selected cases [8]. Several suggestions have been reported in order to define the criteria for ampullectomy such as the tumor's stage, nodal involvement, differentiation, as well as the patients' age and concomitant illness [9,10]. Some authors suggest that all patients with ampullary tumors, even benign ones, should undergo radical resection, citing problems such as a high incidence of malignancy in ampullary villous tumors, difficulty in excluding malignancy with preoperative biopsy, an increased tendency for these lesions to recur after local excision, as well as questions concerning its adequacy as a cancer operation [11-13]. In contrast, other authors believe that local resection is an acceptable form of treatment with satisfactory survival rates in selected patients and markedly decreased morbidity and mortality rates compared with radical resections [10,14,15].

We present our experience with patients who underwent local excision of ampullary tumours. Indications, surgical technique, outcome, and long-term results are discussed with the purpose of drawing attention to the procedure as a possible curative treatment for selected ampullary tumours.

## Methods

From 1990 to 2004, 20 patients suffering from ampullary tumors were treated in our Department with local excision. Twelve (60%) patients were men and 8 (40%) women with mean age 68.5 years (range: 47–79 years). Twelve (60%) patients had carcinomas and eight (40%) had adenomas. Only lesions confined to the ampulla or clearly invading the surroundings tissues from the ampulla, were designated as ampullary carcinomas.

The patients presented with obstructive jaundice, upper abdominal pain, weight loss, nausea, vomiting, pancreatitis, and pruritus either as a single symptom or in combination. The preoperative diagnostic evaluation included estimation of liver function tests (LFTs) and plasma amylase levels, and standard imaging investigations which included conventional ultrasonography (US) scan, computed tomography (CT) scan and endoscopic retrograde cholangiopangreaticography (ERCP) with multiple biopsies taken from the ampulla of Vater. During operation, resection margins were evaluated by frozen section. No patient in the study received adjuvant chemotherapy or radiotherapy.

All the patients who pre or intraoperatively were diagnosed as carcinoma were considered suitable for ampullectomy according to the following criteria: 1) lesion less than 2 cm in diameter, 2) pT1 cancer 3) well or moderate differentiated tumors without nodal involvement. Besides, local excision was the preferable treatment when the patient's concomitant medical illness or age contraindicated a major operation such as PD. All pre or intraoperatively diagnosed benign lesions were considered suitable for ampullectomy, as well. If preoperative ERCP revealed involvement of the distal bile duct or pancreatic duct greater than 1 cm, a PD was considered rather than a local excision.

All patients underwent regular 3 monthly follow-up examinations for the first year, on a 6-month basis for the following 4 years and annually thereafter. Follow-up included clinical examination, blood tests (CA 19-9, LFT), abdominal ultrasound, and chest radiography. Duodenoscopy and ERCP were performed at 6 monthly intervals for two years and then once a year, and earlier if the patient had symptoms.

### Local resection technique

The abdomen was explored through a subcostal or midline incision. After a Kocher maneuver for the mobilization of the second part of the duodenum, the latter was opened by a 4–5 cm "antimesenteric" longitudinal incision. Stay sutures were placed in the duodenal wall circumferentially, and bile and pancreatic duct were canulated with a Fogarty catheter. Then, the normal duodenal mucosa surrounding the ampullary tumor was injected with saline containing 1 to 100,000 epinephrine. Once the identification of the ducts had been accomplished, a circumferential resection of duodenal mucosa to a depth necessary to excise the tumor was undertaken. Margins of 1 cm were obtained in all directions beyond the gross border of the lesion, in order to obtain free margins resection. Frozen section from the tumor and the surrounding tissues was performed to confirm or not tumor's malignancy and to ensure negative margins respectively. Besides, a routine lymph node dissection was performed in patients with ampullary cancer before local excision, which included the supraduodenal as well as anterior and posterior lymph nodes of the pancreatic head. The specimens were also sent for frozen section. If the pathological results did not meet the criteria for a potential curative local resection (pT1 stage, well differentiation, negative lymph nodes) a PD operation was considered, if medically fit, otherwise the ampullectomy was continued. Because bile and pancreatic ducts were transected a reconstruction procedure was essential to ensure adequate billiary and pancreatic drainage and to repair the transduodenal defect. Reconstruction was accomplished by approximating the common walls of the pancreatic and bile ducts that eventually were sutured together on the duodenal wall. Thereafter, the ducts were probed with billiary dilators to ensure appropriate size. A diameter of 6 to 8 mm for the bile duct and 4 to 5 mm for the pancreatic duct were obtained, assuming that scarring will reduce these diameters by 50%. After the establishment of an adequate duct patency the duodenotomy was closed transversely.

## Results

All 20 patients were clinically symptomatic at the time of surgery. The most common symptom for those with adenoma was abdominal pain (68%), whereas nausea and vomiting were second in frequency (38%). Acute pancreatitis was present in 1 of 8 patients with benign lesions. Obstructive jaundice (69%) was the most common presenting symptom among those with malignancy. Abdominal pain (47%), weight loss (38%), and pruritus (30%) were also common. Jaundice and pruritus were more prevalent in those with carcinoma, whereas pancreatitis was more common in patients with benign lesions. The median time of the patients' symptoms was 8 weeks in the adenoma group and 5 weeks among the patients with malignancy.

All the patients of the study underwent preoperative (US) scan, (CT) scan and ERCP with multiple biopsies taken from the suspicious area. ERCP showed that in 14 patients the tumor was localized in the ampulla, whereas in remaining 6 was hidden juts behind the orifice of the ampulla. Duodenoscopy with biopsies failed to reveal malignancy in 10% of the patients. Two of the patients with benign preoperative biopsies had adenocarcinoma detected at the time of the frozen section, which was further confirmed by the final pathologic analysis. In addition to preoperative biopsy all the patients had frozen section (FSE) analysis of the operative specimen. FSE accurately predicted the final histology in all patients. The combination of preoperative and frozen section biopsies was 100% accurate for the diagnosis of adenoma or adenocarcinoma. In none of the patients of our study, surgical margins were found to be positive by frozen section examination. Besides, in all cases, negative frozen sections were also negative for malignancy in the final pathological report, as well.

The median size of the tumors, as measured by the pathologist, was 1.3 cm (range: 0.7–1.8). The carcinomas were classified as pT1 with well or moderate differentiation (G1 or G2) in 10 patients with malignancy, and as pT2 tumors with moderate and low differentiation in the remaining two. There was no lymph node metastasis in any of these patients. PD was considered for the two patients with pT2 tumors. However, concomitant defects such as pulmonary, cardiac and vascular disease precluded safe performance of the PD.

In the early postoperative period, there were no in hospital deaths or any major postoperative complication associated with the procedure. Only two minor complications were recorded, one wound infection and one case of postoperative pneumonia.

**Figure 1:**
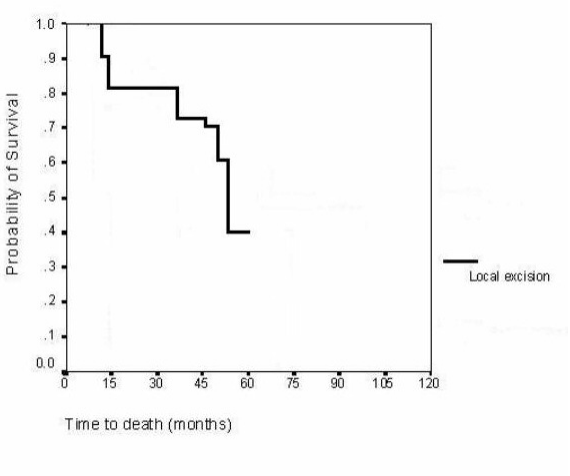
Time to death for patients who underwent local excision (Kaplan – Meier statisticall analysis). The 3 and 5-year survival rates for the patients with adenocarcinoma was 75% and 33,3% respectively.

Median follow up was 85 (6–180) months. During follow-up period, all patients with adenoma are alive without any sign of tumor recurrence. Follow-up was closed for each patient with ampullary carcinoma after a follow-up period of five years unless death occurred during this time. Five patients have died during follow-up and all but two died because of recurrence. Three patients that died due to recurrence include the patients (n = 2) with pT2 carcinomas. Finally, 3 patients are alive at 6 months, 3 and 4 years after the operation respectively. Following local excision of the ampullary cancer in 12 patients, the survival rate at 3 years was 75% (9 patients) and 33.3% (4 patients) at 5 years. In the patients with ampullary cancer that achieved 5-year survival, the resection was R0, the tumor was graded as pT1, N0, M0 and, moreover, it was well differentiated. The probability of survival is shown in figure 1. The median hospital stay was 8 (7–10) days.

## Discussion

Neoplasms of the ampulla of Vater belong to the group of periampullary tumors, which also includes tumors originating from the pancreas, the common bile duct or the duodenum [16,17]. The benign tumors of the ampulla are rare. Among these the most common recognized is adenoma [18], whereas the other benign types (lipomas, neuromas, etc.) are very unusual [19]. Malignant tumors of the ampulla are also rare and adenocarcinoma represents the most common pathological variety. Adenomatous tissue is found in 80% of the adenocarcinomas [20], suggesting that the malignant tumors arise from adenomas. According to this observation, there is little argument over the necessity of adenomas resection, while there is no doubt regarding the resection of malignant tumors, if it is possible.

Controversy exists over how to manage these lesions. Pancreaticoduedenectomy is the standard surgical treatment [2-4], however alternative techniques such as local [8,10,14,15] or endoscopic resection [21], have been also used. In this study, we present our experience on the treatment of benign or malignant lesions of the ampulla using local resection. In our Unit, endoscopic excision was not used due to lack of relevant experience.

The accurate diagnosis and characterization of the ampullary tumors is, undoubtedly, essential, in order to select the appropriate patients for a potentially curative local resection. Conventional techniques, such as ultrasonography and computed tomography (CT), have been used for this reason, but they have been not found sensitive enough in detecting ampullary lesions and, therefore, should not be relied upon for initial diagnosis [22]. There are studies reporting that CT detects the lesion in only 20% of patients [11,22]. However, these methods may offer useful information that would add to the diagnosis and characterization of the tumor. In particular, the dilatation of the pancreatic or common bile duct can be seen in ultrasonography, whereas CT is able to detect metastatic tumors. In three patients of our study the common bile duct was found dilated, whereas none of twelve patients with adenocarcinoma presented with metastatic disease.

The diagnosis of an ampullary tumor may be made by direct visualization using ERCP. ERCP is also helpful in identifying the extent, size, and gross appearance of the tumor, whereas gives the ability of taking endoscopic preoperative biopsies. These biopsies are considered the primary mean for the histological characterization of the lesions. However, they have a variable rate of accuracy, although the fact that the region of the ampulla is easily accessible for such interventions. Some authors have reported false negative biopsies, ranging from 25% to 60% [14,23,24], whereas others have found remarkably lower rates such as 11,7% [25]. These conflicted results led to a consensus that malignancy cannot be reliably excluded based on endoscopic biopsy alone. In our series, endoscopic preoperative biopsy showed similar lack of accuracy, as it failed to detect the malignancy in 17% of cases. However, this diagnostic problem can be ameliorated with the use of frozen section analysis intraoperatively. It has been suggested that FSE is a useful adjunct in distinguishing adenoma from adenocarcinoma, predicting accurately the final histology in all patients [25]. A recent study, reported sensitivity of 85% and specificity of 100% for intraoperative frozen section biopsy, concerning adenocarcinoma [25]. Furthermore, frozen section examination of the resection margin during ampullectomy may help to obtain a free resection margin. In our patients, FSE accurately predicted the final histology in all cases, while the combination of preoperative and frozen section biopsies was 100% accurate for the diagnosis of adenoma or adenocarcinoma, which is in accordance with findings reported by other authors [1]. In addition to ERCP biopsies and FSE, another useful adjunct for the preoperative planning is Endoscopic ultrasound (EUS) [10,22]. EUS cannot replace histological evaluation, and thus, a differentiation between an adenoma and a pT1 carcinoma is not possible. However, EUS may be extremely useful as the most accurate method to determine tumor stage and select patients for local excision. It is stated that EUS can easily differentiate a grossly infiltrating tumor from an early cancinoma [10]. EUS is also reported to delineate clearly the layered structures of the duodenal wall in the ampullary region and to diagnose accurately the presence and extent of tumor invasion into duodenum or common bile duct wall and the involvement of the pancreas [22]. Underestimating the depth of invasion occurs rarely while over staging is reported to occur in one third of pT1 tumors [10]. EUS was not included in our diagnostic tools due to lack of experience and relevant equipment. It seems that EUS might have been very helpful in preoperative identification of the patients who met the criteria for local excision in our study.

Based on these diagnostic procedures, 8 out of 20 patients in our series were proved to have an adenoma, while the remaining had an adenocarcinoma. In all these patients, a local resection technique was applied. In benign lesions, there are many reports suggesting that local excision is an inadequate treatment, stating problems, such as a high incidence of malignancy in benign ampullary tumors and an increased tendency of these lesions to recur after local resection [11-13]. Galandiuk *et al *[23], reported a local recurrence rate of 42% among 13 patients who underwent local excision for villous tumors. In a more recent study, Farnell *et al *[26] found a recurrence rate of 32% at 5-years and 43% at 10 years in 53 patients with villous tumors, who underwent transduodenal submucosal resection. This high rate of recurrence was attributed to narrow margins for lesions close to the ampulla, which resulted to inadequate free margin resection. On the other hand, proponents of local resection emphasize the importance of obtaining adequate margins and report low recurrence rates [10,14,15]. Rattner *et al *[10], reported no local recurrences at a mean follow-up of 29 months, including two patients with invasive carcinoma. In a recent study, 17 out of 18 patients with benign lesions remained disease free for a mean follow-up time of 38 months [27]. Most studies cite low rates of recurrence for benign adenomas, ranging from 11% to 33% [10,13-15,23]. In our series, after a median follow-up of 85 months all patients with adenoma are alive without any sign of tumor recurrence.

Although there are many case reports and a few series on the treatment of ampullary malignancies by local ampullary excision, the criteria used to decide when local excision is suitable for patients with adenocarcinoma are controversial, and not well addressed. We performed local resection to patients with small (less than 2 cm), pT1 tumors, well or moderate differentiated carcinomas, as well as in cases with advanced adenocarcinomas (pT2), which were unfit for PD, due to concomitant illness. There was no lymph node metastasis in any of these patients. To achieve operative curability with ampullectomy, two criteria seem that should be fulfilled: no lymph node metastasis and a free resection margin. As we mentioned before, a free margin resection can be achieved in most cases by an adequate local resection technique, in combination with intraoperative biopsies. On the other hand, lymph node metastasis, perineural invasion and lymphovascular invasion have been identified as important factors affecting the survival and the recurrence after local resection [28]. Therefore, the selection of ampullary cancers without lymph node metastasis preoperatively is essential for a curative local resection. However, preoperative imaging modalities have limitations in terms of diagnosing regional lymph node metastasis and even EUS has been found to be inadequate to predict accurately the presence or absence of lymph node metastasis [29,30]. Thus, it became essential to identify clinicopathologic factors able to predict lymph node metastasis before operation. Bottger and Junginger [5] reported that lymph node metastasis were not found in small, or T1 tumors, or in tumors with well differentiated histology. Similar results have been also reported from other authors [9,28,30,31]. Rattner *et al *[10], recommended ampullectomy for T1 cancer, and Beger *et al *[14] for Tis or T1, N0, M0 cancer with well or moderate differentiation. These findings suggest that small tumors, staging as Tis or T1 with well differentiation, appears to be an essential condition for local resection.

Although the fact that the vast majority of the ampullectomy studies include limited number of patients, their results in regard to morbidity, mortality and long term survival are encouraging and thus, cannot be ignored. Rattner *et al *[10], reported nil morbidity and mortality rates after local resection, while other authors, found mortality rates ranging from 0% to 25% [32-36]. Similar results were also reproduced in our study. We had nil morbidity and mortality rates after ampullectomy, even in the group with adenocarcinoma patients. Attempting to explain these rates we must underlie the advantages of local resection as surgical technique. Local resection is a simple technique, which can be performed safely, requiring significantly less time and with decreased blood loss. This is translated into a decrease in the number of patients requiring perioperative blood transfusions. Blood transfusion has been reported to be related with poor prognosis in patients undergoing resection for ampullary cancer [28]. Additionally, the in-hospital length of stay after ampullectomy is relatively low (10 days) [10,25], leading to a decreased chance of developing complications resulting from hospitalization. In regard to long-term survival the results are encouranging. Shutze *et al *[37], reviewed 15 series totalling 520 patients with a combined survival of 40%. Six other series totalling 68 patients report a 5-year survival rate of 40% [1,33-35,38,39]. In our study, which included a small number of patients, following local excision of the ampullary cancer, the survival rate at 3 and 5 years was 75% and 33,3% respectively.

## Conclusion

In our series, local excision was a safe option, associated with satisfactory long-term survival rates in patients with benign lesions and in those with malignant ones, who had small (<2 cm), pT1, well differentiated ampullary tumours without nodal involvement.

## Competing interests

The author(s) declare that they have no competing interests.

## Authors' contributions

**DH**. performed a number of operations, offered patients' data, involved in drafting the manuscript and critically revised and approved the manuscript.

**ZE **participated in the operations, performed the statistical analysis and involved in drafting the manuscript.

**KIE **collected patients' data and involved in drafting the manuscript.

**PMG **participated in the operations and data collection.

**SN**. participated in the operations and data collection.

**KI **performed a number of operations and offered patients' data.

**BD **critically revised and gave approval to the final version of the manuscript
